# Analysis of Intracellular and Extracellular Selenium Concentrations: Differences According to Training Level

**DOI:** 10.3390/nu14091857

**Published:** 2022-04-29

**Authors:** Víctor Toro-Román, Ignacio Bartolomé, Jesús Siquier-Coll, María C. Robles-Gil, Diego Muñoz, Marcos Maynar-Mariño

**Affiliations:** 1 School of Sport Sciences, University of Extremadura, Avenida de la Universidad s/n, 10003 Cáceres, Spain; vtoro@unex.es (V.T.-R.); ibartolomesa@upsa.es (I.B.); mcroblesgil@unex.es (M.C.R.-G.); mmaynar@unex.es (M.M.-M.); 2 Department of Didactics, Faculty of Education, Pontifical University of Salamanca, C/Henry Collet, 52–70, 37007 Salamanca, Spain; 3 SER Research Group, Center of Higher Education Alberta Giménez (Affiliated to Comillas Pontifical University), 07011 Palma de Mallorca, Spain; jesussiquier@cesag.org

**Keywords:** training, selenium, trace mineral elements, soccer

## Abstract

Trace mineral element concentrations are under homeostatic control. Selenium (Se) is a very important micronutrient for the antioxidant and immune system. Se metabolism could be modified due to physical training. This research aimed to analyze the extracellular (plasma, urine and serum) and intracellular (platelets and erythrocytes) concentrations of Se in athletes and to compare it with subjects with low levels of physical training. Forty young men divided into a control group (CG; *n* = 20; 19.25 ± 0.39 years) and a training group (TG; *n* = 20; 18.15 ± 0.27 years) participated in this study. The TG was formed by semi-professional soccer players. The analysis of Se was determined by inductively coupled plasma mass spectrometry. The TG obtained higher values of maximum oxygen consumption and muscle percentage (*p* < 0.05). The TG showed reduced absolute (*p* < 0.01) and relative (*p* < 0.05) Se concentrations in erythrocytes and platelets in comparison to CG. Trace element assessments should not be limited only to extracellular compartments as there could be deficiencies at the intracellular level.

## 1. Introduction

Selenium (Se) is an essential trace mineral element (TME) in humans but is toxic when consumed in excessive amounts [1]. It was discovered in 1817, and its physiological importance in the body was determined in the mid-1950s [2]. It is usually sourced from seafood, peas, lentils, beans, whole grains, organ meats, dairy products and vegetables [3]. An adult contains 20 mg of Se, which is stored mainly in organs and tissues in different amounts: 30% in the liver, 30% in muscle, 15% in the kidney, 10% in plasma and 15% in other organs [1,4,5]. Se is present in nature and in organisms in organic or inorganic forms. The main organic forms are selenomethionine and selenocysteine, while the inorganic forms are selenite, selenide, selenate and Se [3].

It is incorporated into proteins in the form of selenocysteine and selenomethionine, forming selenoproteins [6]. Se is an important element of selenoproteins that are involved in redox catalytic activity and structural and transport functions [6]. Se is linked to antioxidant defense, immune function, thyroid hormone synthesis, testosterone metabolism, DNA structure, vitamin E modulation, anticancer and inflammatory processes and muscle performance [3,7].

Among the different selenoproteins, the enzymes glutathione peroxidase (GPx) and glutathione reductase (GR) are well known in sports. These enzymes comprise the glutathione redox cycle (GSH), an essential antioxidant system in the body [4,7,8].

TME concentrations are usually under rigorous homeostatic control [9]. However, Se metabolism could be modified during physical exercise [4]. Previous studies researching Se concentrations have mainly been based on the effects of the intake of Se-rich supplements [10,11,12,13,14]. Research concerning the influence of physical exercise on Se concentrations in different compartments is limited [15,16,17,18]. Moreover, previous investigations have analyzed up to three compartments to assess Se status, with no studies evaluating Se concentrations in more compartments simultaneously.

There are several compartments to assess Se status. It could be measured directly in plasma, serum, blood, urine, hair, nails and in tissues such as the kidney and liver [3]. Se can be measured indirectly by GPx activity [3]. The evaluation of Se status in an isolated compartment does not seem to be adequate to obtain a complete assessment [19]. Plasma or serum Se could reflect the short-term status, whereas erythrocyte Se could indicate the long-term status [20]. Previously, Combs Jr. (2015) reported the need for additional biomarkers to assess Se status [21]. One of the intracellular compartments studied to a lesser extent is platelets. It is known that platelet Se concentrations are considerable [22,23]. Kiem [24] concluded that an analysis in platelets could be useful to detect deficiencies in Se intake. Similarly, the levels of GPx in platelets appears to be a more sensitive indicator of Se intake compared to GPx in erythrocytes [20,25]. This could be related to the shorter half-life of platelets, of 8 to 14 days, compared to 120 days for erythrocytes [23]. Therefore, based on the half-life, an analysis in platelets could provide more updated information on intracellular Se concentrations. Nevertheless, intraplatelet Se concentrations in this population have not been studied. 

Given the existing deficiencies in the assessment of Se status in one compartment and the effect of physical exercise on TME status, the objective of the study was to analyze intracellular (erythrocytes and platelets) and extracellular (plasma, urine and serum) concentrations in athletes and to compare them with subjects with low levels of physical exercise.

## 2. Materials and Methods

The participants and methodology were similar to that reported by Toro-Román et al. [19,26]. All tests were performed in the morning under similar atmospheric conditions (21–24 °C and 45–55% relative humidity). In the TG, training volume and intensity were reduced for the two days prior to the assessments.

### 2.1. Participants

Participants were informed of the purpose of the study and signed a consent form. The protocol was approved by the Bioethics Committee of the University of Extremadura (Cáceres, Spain) following the guidelines of the Helsinki ethical declaration for experimentation on human beings (13/2021). All of them fulfilled the inclusion criteria: being a man; not having had any injury or disease during the research or at least 6 months before the study and not following any special diet or taking any vitamin/mineral supplements, specific supplementation or over-the-counter medication.

A total of 40 men, divided into a control group (CG; *n* = 20; age = 19.25 ± 0.39 years; weight = 73.45 ± 9.04 kg; height = 1.79 ± 0.06 m) and training group (TG; *n* = 20; age = 18.15 ± 0.27 years; weight = 68.59 ± 4.18 kg; height = 1.76 ± 0.04 m) participated in this research. All the participants had been living in the area of Caceres (Spain) for at least 24 months before the beginning of the research. The TG consisted of semi-professional soccer players with a regular training plan of 10 h/week and at least five years of experience in high-level competitions. The CG consisted of healthy young male sports science students who did not participate in physical exercise regularly and had not followed any specific training plan in the previous 6 months. On the day of the assessments, the International Physical Activity Questionnaire Short Form (IPAQ-SF) Spanish version [27] was used to assess physical activity. A nutritional evaluation of macronutrients, water and Se intake was also carried out following the guidelines of Toro-Román et al. [19,26]. Participants completed a dietary questionnaire to ascertain nutritional intake on 4 days (three pre-assigned weekdays and one weekend day). On each day, all participants recorded the amount (in grams) of each food.

### 2.2. Anthropometric Measurements

A wall-mounted stadiometer (Seca 220, Hamburg, Germany), a calibrated electronic digital scale (Seca 769, Hamburg, Germany), a Holtain© 610ND (Holtain, Crymych, UK) skinfold compass, a Holtain© 604 (Holtain, Crymych, UK) bone diameter compass and a Seca© 201 (Seca, Hamburg, Germany) brand tape measure were used for the anthropometric assessments. All measurements were made by the same operator. The equations of the Spanish Group of Kinanthropometry [28] were used to calculate the muscle and fat percentage. The anthropometric measurements obtained were height, weight, bone diameters (bistyloid, humeral biepicondyle and femoral biepicondyle), skinfolds (abdominal, suprailiac, subscapular, tricipital, thigh and leg) and muscle perimeters (relaxed arm and leg).

### 2.3. Physical Performance Test

To evaluate performance variables, a maximal incremental exercise test to exhaustion was performed after blood sampling. The test was performed on a treadmill (Ergofit Trac Alpin 4000, Pirmasens, Germany), equipped with a gas analyzer (Geratherm Respiratory GMBH, Ergostik, Ref 40.400, Corp Bad Kissingen, Geschwenda, Germany), and subjects wore a Polar heart rate monitor (Polar^®^ H10, Kempele, Finland). All tests were performed approximately 2 h after a free breakfast.

Participants performed a progressive warm-up for 15 min, finishing at the initial speed of the test. The protocol consisted of running in incremental stages until voluntary exhaustion, starting at an initial speed of 8 km/h for the CG and 10 km/h for the TG, and increasing by 1 km/h every two minutes, with a steady 1% slope.

### 2.4. Sample Collection and Selenium Determination

A total of 12 mL of venous blood was drawn from each subject. For serum, a 5 mL blood sample was collected in a polypropylene tube and then centrifuged at 3000× *g* rpm for 15 min. For plasma, a 5 mL blood sample was collected in a polypropylene tube with ethylenediaminetetraacetic acid (EDTA) and then centrifuged at 1800× *g* rpm for 8 min at room temperature. The platelet-rich plasma (PRP) obtained was collected in a metal-free polypropylene tube and centrifuged for 15 min at 3000× *g* rpm. The plasma was aliquoted into an Eppendorf tube and allowed to stand at −80 °C until further analysis. One milliliter of pure water was added to the tube of platelet concentrate and stored at −80 °C. Erythrocytes were removed from the remaining blood and washed three times with 0.9% sodium chloride (NaCl). They were then aliquoted into Eppendorf tubes and stored at −80 °C until biochemical analysis. Finally, the first morning urine sample obtained from all subjects, while fasting, was collected in polyethylene tubes previously washed with dilute nitric acid and frozen at −80 °C until analysis.

The remaining 2 mL of blood was used for red blood cell and platelet determination using an automatic cell counter (Coulter Electronics LTD, Model CPA; Northwell Drive, Luton, UK).

Se determination in the different compartments (plasma, serum, urine, erythrocytes and platelets), sample preparation and sample analysis were conducted following the methodology of Toro-Román et al. [19,26] (see previous references for details) using an inductively coupled plasma mass spectrometry (ICP-MS) (NexION 300D; PerkinElmer; Inc.; Shelton, CT, USA).

### 2.5. Statistical Evaluations

Statistical analyses were carried out with IBM SPSS Statistics 22.0 for Windows (SPSS Inc., Chicago, IL, USA). A *p* < 0.05 was considered statistically significant. The normality of the distribution of variables was analyzed using the Shapiro–Wilk test. A student’s *t*-test was used to compare the concentrations between both groups. Effect sizes (ES) were calculated using Hedge’s g [29]. The equation for calculating ES was obtained from the manuscript by Tomczak and Tomczak [30]. ES of 0.2, 0.5 and 0.8 were considered small, moderate and large, respectively [30].

## 3. Results and Discussion

For a better understanding, the results and discussion sections will be unified. Table 1 details the characteristics of the participants. There were significant differences in muscle percentage, fat percentage, expiratory volume, maximal oxygen consumption (VO_2max_), physical activity levels and resting heart rate (*p* < 0.05).

Table 2 shows that there were no differences between groups regarding energy, macronutrients, water and Se intake.

Excessive Se intake could be detrimental. However, inadequate amounts could increase exercise-induced oxidative stress [31]. A study conducted in 553 elite athletes observed deficiencies in Se intake [32]. Previously, it has been reported that athletes require higher Se intakes to increase the functionality and activity of antioxidant systems [33]. In addition, the adaptive response of the endogenous antioxidant system to physical training is also dependent on nutritional factors [13]. In this study, the participants of both groups ingested Se above the Dietary Reference Intakes (DRI = 55 μg/day) [34] with no differences. Therefore, it is possible that the observed changes in Se concentrations could be due to the influence of physical training.

Table 3 reflects the values of erythrocytes and platelets in both groups. There were no differences.

Table 4 shows the extracellular concentrations of Se. There were no differences in any of the compartments analyzed.

Studies assessing Se concentrations in general populations have used direct (plasma, serum and erythrocytes) and indirect (plasma, erythrocyte and platelet GPx concentrations) methods, mainly in isolation and after Se supplementation [35,36]. Regarding indirect measurements, previous authors reported that GPx activity is not suitable as a marker of Se status [37]. In terms of direct measurements, plasma Se is the most widely used in the literature, although it is not generally considered an ideal biomarker of Se status. [35] The sensitivity of plasma Se to variations in Se status is unclear [23]. Moreover, serum Se concentrations could reflect Se status with health effects [20]. However, the interpretation of the results could be difficult in participants with a systemic inflammatory response [38]. Otherwise, erythrocyte Se concentrations could indicate long-term Se status due to a longer half-life (approximately 120 days). In addition, it is known that Se incorporation occurs during erythrocyte synthesis [20,39]. As mentioned above, the assessment of platelet Se status could provide more up-to-date information due to the short half-life (approximately 8 to 14 days) [23]. Short-term variations in Se status only appear to influence recently synthesized cells, since Se incorporation occurs in bone marrow cells [23]. Therefore, based on the fact that each compartment could provide information on Se status at different times, it seems necessary to assess extracellular and intracellular compartments simultaneously to obtain complete information on Se status. The concentrations found in the present study are within the ranges reported in previous studies using similar techniques [15,40,41,42,43].

No changes in extracellular Se concentrations were observed in this research. Rousseau et al. [44] and Sanchez et al. [45] did not observe differences when comparing young people and sedentary or physically active adults in plasma, like this study. However, Tessier et al. [46] reported lower concentrations after 10 weeks of aerobic training. In contrast, Margaritis et al. [33] found higher concentrations in active people. Concerning serum levels, Maynar et al. [16,17] observed lower concentrations in the TG, to a greater extent in aerobic athletes. Finally, Maynar et al. [16,47] reported lower urinary levels of Se in athletes.

Table 5 shows the intracellular concentrations of Se. The TG showed lower Se concentrations in erythrocytes and platelets, in absolute and relative values (*p* < 0.05).

The aim of the present study was to analyze the extracellular (serum, plasma and urine) and intracellular (erythrocytes and platelets) concentrations of Se in athletes and to compare it with subjects with low levels of physical training. We found lower intracellular Se concentrations in the TG, with no differences in extracellular concentrations. To our knowledge, this is the first study to simultaneously analyze five compartments to assess Se status. In addition, no study in athletes analyzing Se concentration in platelets has been reported.

According to the results obtained, these results could reflect deficiencies in Se status in the TG both in the long and short term. Se is transported to all body tissues and organs, including red blood cells [4], where it is stored after being absorbed. Stored Se is utilized when Se intake is too low for selenoprotein synthesis, reducing Se reserves [48]. As reflected by intracellular concentrations, despite similar intakes, the TG could be deficient in both long-term and short-term Se intake. Therefore, it is necessary to increase Se intake in athletes performing regular physical training, as previously reported [33].

The data reported in this study are in agreement with those reported by Maynar et al. [15] in erythrocytes from athletes. Furthermore, the authors determined an inverse relationship between the training status and the concentration of Se in erythrocytes (r= −0.275; *p* = 0.021). Likewise, Pograjc et al. [49] reported lower Se levels in erythrocytes after 3 months of military training. Nonetheless, plasma Se values and GPX activity in erythrocytes increased. To our knowledge, no studies on Se platelet concentrations in athletes have been reported. Reduced Se intakes were associated with a lower Se in erythrocytes and platelets in children [50] and sheep [25]. On the same point, the rise in Se intake was more reflected in platelets compared to serum and whole blood, confirming that intraplatelet assessment of Se was more effective than in serum and whole blood [51] and that intraplatelet Se evaluation could provide information on short-term Se status [23].

Physical exercise causes an increase in the inflammatory response and oxidative stress [52]. As mentioned above, Se is an essential cofactor of different selenoproteins, with multiple roles in anti-inflammatory and antioxidant functions [7]. Physical training generates adaptations of the components of the antioxidant system [46,53]. Se incorporation into selenoproteins and its distribution in tissues is thought to be different between athletes and sedentary subjects [33]. The fall in intracellular Se concentrations could indicate that part of Se is transferred to tissues due to the consequences of physical training [15,49]. Se could be released into the extracellular compartment to counteract inflammation and the action of free radicals and lipid peroxides caused by physical exercise by increasing selenoprotein activity and synthesis. In this regard, reduced extracellular concentrations of Se correlated with increased muscle damage and inflammation [54], which could justify the intracellular outflow of Se.

Pograjc et al. [49] reported that maximal plasma GPx activity is reached at blood Se levels above 95 ng/g (1.27 μmol/L or 100 μg/L). According to the results obtained and based on the above data, the TG would not reach plasma Se concentrations sufficient to have maximal GPx activity. Therefore, Se could also enter the extracellular compartments to increase plasma selenoprotein activity [17].

This study has some limitations: (i) the small single-sex sample and (ii) the lack of complementary Se data, such as GPx concentrations, GPx activity, GSH and GR. Future research could incorporate a group of females, as well as evaluate the influence of age on Se concentrations combined with the determination of selenoproteins.

## 4. Conclusions

Despite similar intakes, TG had lower Se concentrations in erythrocytes and platelets. However, there were no differences in extracellular compartments. Se leaves intracellular compartments due to inflammation and oxidative stress caused by regular physical training.

It does not appear to be appropriate to analyze only the extracellular compartments when assessing Se status, since there could be a deficit in intracellular compartments as observed in the present study. The extracellular and intracellular evaluation of Se should be performed in athletes who regularly perform physical training.

## Figures and Tables

**Table 1:** Characteristics of participants.

Parameters	CG (*n* = 20)	TG (*n* = 20)
Muscle (%)	44.22 ± 5.71	49.03 ± 2.56 *
Fat (%)	15.64 ± 5.78	9.32 ± 2.76 *
VO_2max_ (mL/kg/min)	45.61 ± 4.95	61.02 ± 4.35 **
VE_max_ (L/min)	88.34 ± 11.18	120.56 ± 18.79 **
Resting heart rate (bpm)	67.31 ± 6.49	54.41 ± 5.29 *
Maximum heart rate (bpm)	189.3 ± 7.1	193.8 ± 6.5
Physical activity (MET-hours/weekly)	27.36 ± 4.45	56.13 ± 6.21 **

VO_2max_ = maximal oxygen consumption; VE_max_: maximal expiratory ventilation; MET = Metabolic equivalent task; TG = Training group; CG = Control group; TG = Training group; * *p* < 0.05; ** *p* < 0.01.

**Table 2:** Nutritional intakes of participants.

Parameters	CG (*n* = 20)	TG (*n* = 20)
Energy (Kcal/day)	2112.34 ± 345.78	2456.16 ± 504.11
Water (L/day)	1.145 ± 0.241	1.421 ± 0.356
Carbohydrates (g/kg/day)	3.11 ± 1.28	3.98 ± 1.78
Proteins (g/kg/day)	1.25 ± 0.37	1.44 ± 0.41
Lipids (g/kg/day)	1.51 ± 0.47	1.64 ± 0.31
Se (μg/day)	97.33 ± 14.85	101.25 ± 16.41

CG = Control group; TG = Training group; Se = Selenium.

**Table 3:** Erythrocytes and platelets values.

Parameters	CG (*n* = 20)	TG (*n* = 20)	ES
Erythrocytes (cell 10^12^ /L)	4.81 ± 0.72	4.76 ± 0.89	0.14
Platelets (cell 10^9^ /L)	190.23 ± 67.13	198.35 ± 60.51	0.17

CG = Control group; TG = Training group; ES = Effect size.

**Table 4:** Plasma, serum and urinary concentrations of Se.

Parameters	CG (*n* = 20)	TG (*n* = 20)	ES
Plasma (μg/L)	91.16 ± 9.98	90.13 ± 8.56	0.07
Serum (μg/L)	80.63 ± 8.14	76.42 ± 7.47	0.36
Urine (μg/L)	31.44 ± 11.18	28.68 ± 5.78	0.13

CG = Control group; TG = Training group; ES = Effect size.

**Table 5:** Se concentrations in erythrocytes and platelets.

Parameters	CG (*n* = 20)	TG (*n* = 20)	ES
Erythrocytes (μg/L)	139.28 ± 23.64	102.25 ± 25.46 **	1.08
Erythrocytes (pg/cell10^−6^)	28.73 ± 3.69	21.75 ± 5.67 **	0.86
Platelets (μg/L)	9.86 ± 2.91	6.90 ± 2.11 **	0.67
Platelets (pg/cell 10^−3^)	0.051 ± 0.011	0.034 ± 0.009 *	0.52

CG = Control group; TG = Training group; ES = Effect size; * *p* < 0.05; ** *p* < 0.01.

## Data Availability

Not applicable.
